# Give and take? The unexpected benefits of public engagement as an end-in-itself social practice in the Aberdeen Children of the 1950s cohort study.

**DOI:** 10.23889/ijpds.v7i3.2008

**Published:** 2022-08-25

**Authors:** Krzysztof Adamczyk, Shantini Paranjothy, Marjorie Johnston

**Affiliations:** 1 University of Aberdeen; 2 NHS Grampian

## Objectives

Public engagement in cohort studies is instrumental to recruitment and retention. However, instead of framing engagement as a means to an end, we advocate for an end in itself approach that attempts to meaningfully share the shaping of the narrative of the cohort with its members.

## Approach

Through an annual postal newsletter, we recruited five cohort members to lead the creation of a digital exhibition about the Aberdeen Children of the 1950s cohort. Our co-curators worked with us and a museum curator to design a narrative-driven overview of the cohort and its members’ lives. Additionally, we sent out a questionnaire (by email) asking cohort members about future research directions (n = 741) including a question: “Are there any other aspects of life during the pandemic that you think should be researched?”. Both activities aimed to contribute qualitative textual material to be drawn upon in this largely quantitative study.

## Results

The digital exhibition was unexpectedly well received, becoming the most visited in the university’s collection. Co-curators emphasized the socio-historical embeddedness of the cohort, sharing experiences of growing up in times of Aberdeen’s booming oil economy. We garnered media attention in newspaper articles and interest from TV producers. Research enabled by the cohort featured in two documentaries, with two cohort members appearing in one.

The questionnaire yielded a response rate of 61%, with just under half providing answers to the open-ended question. Participants found mental health to be of the highest priority to research, contextualizing their rationales in free-text answers (27 mentions). Some of the most common themes included: comparisons with the typhoid epidemic in Aberdeen in the 1960s (9 mentions), and financial struggles (9 mentions).

## Conclusion

Public engagement as an end-in-itself social practice is a powerful way to give agency to research participants. Qualitative contextual material can help researchers to ground their quantitative analyses in historical contexts housing populations which may be essential for interpretation and translation of findings to meaningful policies.

